# Age-Related Developmental and Individual Differences in the Influence of Social and Non-social Distractors on Cognitive Performance

**DOI:** 10.3389/fpsyg.2018.00863

**Published:** 2018-06-08

**Authors:** Patricia Z. Tan, Jennifer S. Silk, Ronald E. Dahl, Dina Kronhaus, Cecile D. Ladouceur

**Affiliations:** ^1^Department of Psychiatry, UCLA School of Medicine, Los Angeles, CA, United States; ^2^Department of Psychology, University of Pittsburgh, Pittsburgh, PA, United States; ^3^Community Health Sciences & Joint Medical Program, School of Public Health, University of California, Berkeley, Berkely, CA, United States; ^4^Cambridge Computational Biology Institute & Wolfson Brain Imaging Centre, Faculty of Music and Computer Laboratory, University of Cambridge, Cambridge, United Kingdom; ^5^Department of Psychiatry, University of Pittsburgh School of Medicine, Pittsburgh, PA, United States

**Keywords:** cognitive control, emotional interference, adolescence, emotion regulation, anxiety, temperament

## Abstract

This study sought to examine age-related differences in the influences of social (neutral, emotional faces) and non-social/non-emotional (shapes) distractor stimuli in children, adolescents, and adults. To assess the degree to which distractor, or task-irrelevant, stimuli of varying social and emotional salience interfere with cognitive performance, children (*N* = 12; 8–12y), adolescents (*N* = 17; 13–17y), and adults (*N* = 17; 18–52y) completed the Emotional Identification and Dynamic Faces (EIDF) task. This task included three types of dynamically-changing distractors: (1) neutral-social (neutral face changing into another face); (2) emotional-social (face changing from 0% emotional to 100% emotional); and (3) non-social/non-emotional (shapes changing from small to large) to index the influence of task-irrelevant social and emotional information on cognition. Results yielded no age-related differences in accuracy but showed an age-related linear reduction in correct reaction times across distractor conditions. An age-related effect in interference was observed, such that children and adults showed slower response times on correct trials with socially-salient distractors; whereas adolescents exhibited faster responses on trials with distractors that included faces rather than shapes. A secondary study goal was to explore individual differences in cognitive interference. Results suggested that regardless of age, low trait anxiety and high effortful control were associated with interference to angry faces. Implications for developmental differences in affective processing, notably the importance of considering the contexts in which purportedly irrelevant social and emotional information might impair, vs. improve cognitive control, are discussed.

## Introduction

Adaptive behavior, for example goal-directed behavior, often requires resisting interference from salient, but task-irrelevant, information while sustaining attention on relevant information. Some degree of interference might be expected given that rapid detection of socially-salient stimuli, notably emotional facial expressions, can be advantageous (Pessoa et al., 2002). However, numerous studies have reported stronger interference to emotional information (e.g., attentional biases) in individuals with anxiety and mood disorders (for a review, see Bar-Haim et al., 2007; Joormann and Quinn, 2014), suggesting that interference from emotionally-salient distractors contributes to affective symptoms. Developmental differences in interference could also be important for understanding why adolescence appears to be a period of increased risk for affective disorders (Paus et al., 2008), in part, because of adolescents' increased sensitivity to social and emotional information (e.g., Casey et al., 2008; Nelson et al., 2016; Schriber and Guyer, 2016). Yet, recent models have also highlighted these years as a period of opportunity, when heightened social and emotional salience promotes the development of complex self-regulatory skills (Crone and Dahl, 2012). Thus, the primary aim of this study was to investigate age-related differences in the influence of distractor stimuli that vary in social (i.e., shapes vs. face) and emotional (i.e., neutral vs. emotional facial expressions) salience across the child, adolescent, and adult years.

Cognitive interference generally refers to the capture of attentional resources by task-irrelevant stimuli that impair cognitive performance. Using “classic” cognitive control tasks such as the Flanker (Grose-Fifer et al., 2013), Stroop and Go-Nogo (Tottenham et al., 2009), several studies have examined age-related differences in interference from distractors that include socially- and/or emotionally-relevant information, including facial expressions or emotional words (e.g., Tottenham et al., 2009; Cohen-Gilbert and Thomas, 2013; Grose-Fifer et al., 2013; Waszczuk et al., 2015). Results from some of these studies report a linear decrease in interference from distractors from childhood into adulthood (e.g., Tottenham et al., 2009; Somerville et al., 2011) that parallels neurodevelopmental evidence for significant maturation in neural systems supporting cognitive control into the adult years (e.g., Bunge et al., 2002; Rubia et al., 2006; Toga et al., 2006). Others instead report a *curvilinear* developmental pattern, with emotional distractors showing the greatest influence on task performance during the adolescent developmental period (e.g., Cohen-Gilbert and Thomas, 2013; Grose-Fifer et al., 2013). This curvilinear pattern has been attributed to a maturational gap between highly-reactive emotion processing and still-maturing cognitive control neural systems (Casey et al., 2008). In other words, in the context of still-maturing prefrontal cortical regions, adolescents are thought to react more strongly to social and emotional information than children and adults. Consequently, adolescents are more likely to show a preferential processing for social and emotional information (e.g., Monk et al., 2003), a preference which is posited to underlie an adolescent-specific peak interference from emotional distractors that increases adolescents' vulnerability to affective disorders.

The variability in findings regarding the development of cognitive interference—and the regulation of such interference—also needs to be considered in relation to task differences. First, tasks often differ in the dimension(s) of cognitive control that have to be engaged for successful performance. For example, one commonly-utilized task—the Emotional Go-Nogo—has an added demand of encoding the emotional (or social) nature of stimuli before, or while, inhibiting a prepotent response. As such, it is difficult to differentiate between difficulty suppressing attentional processing of emotionally-salient distractors and difficulty suppressing a prepotent motor response (e.g., Tottenham et al., 2009). Consequently, such tasks make it difficult to characterize specific age-related differences in a fundamental aspect of emotional interference—the ability to inhibit/shift attention away from an emotional feature of a stimulus. Second, studies have used a wide range of distractor stimuli, including affective words, pictures of emotionally-evocative images, and facial expressions. Because few studies have systematically compared interference specific to non-social/non-emotional (e.g., shapes), neutral-social (e.g., neutral facial expressions or non-social affective scenes), and emotional-social (e.g., emotional facial expressions or affective scenes depicting faces/human interactions) distractors, it is difficult to determine the emotional and/or social dimensions along which interference might vary across different developmental periods. This is an important issue to consider given evidence that emotional valence influences regulation of interference from task-irrelevant information. For example, research on emotional information processing suggests that individuals tend to preferentially attend to threat-related signals such as angry and fearful facial expressions (e.g., Dolan, 2002; Feldmann-Wüstefeld et al., 2011). Indeed, recent developmental examinations of emotional interference found that adolescents (aged 11–19 years) showed greater interference to negative compared to positive and neutral emotional conditions on an emotional Go-Nogo task (Cohen-Gilbert and Thomas, 2013) and to fearful compared to happy faces on an emotional Flanker task (Grose-Fifer et al., 2013).

Third, tasks can also differ in the degree to which emotional distractors are relevant to the task objective. Although the emotional and social content of distractor stimuli are task-irrelevant in traditional tasks used to assess cognitive interference, there is nonetheless variability with regard to other dimensions of task relevancy, notably, spatial location. Distractor stimuli are often presented in a different spatial location from target stimuli, directing attentional resources away from processing task-relevant information (e.g., Grose-Fifer et al., 2013), or as a background stimulus, dividing attentional resources between processing task-irrelevant emotional background information and task-relevant focal information (e.g., Cohen-Gilbert and Thomas, 2013). Task performance could therefore be improved if task-irrelevant information is presented in the field of view or superimposed onto task-relevant information (e.g., Frühholz et al., 2009; Kanske and Kotz, 2011). In such a case, it is reasonable to expect that adolescents might show less cognitive interference from social/emotional distractors than either adults or children because the high saliency of these distractors more strongly engages adolescents and/or directs their attention toward the target stimulus. In short, if increased sensitivity to social and emotional information can serve as either a vulnerability or opportunity for adolescents, it is important to determine the conditions whereby task-irrelevant social and emotional information might support, rather than impair, cognitive performance.

Additionally, no studies have examined interference using dynamically-emerging facial expressions that morph from neutral-to-100% emotional in a community (i.e., emotionally-healthy) sample of youth and adults. Given the dynamic nature of social interactions, tasks using dynamic faces could better mirror the demands of day-to-day socio-emotional information processing. Using such stimuli, recent work has suggested that alterations in patterns of neural functioning underlying interference regulation are important in the development of mood disorders (Perlman et al., 2012; Manelis et al., 2015). The extent to which interference to emotionally-salient social distractors is a vulnerability factor in anxiety disorders can be better understood by determining how this process varies as function of anxiety-related traits. Evidence linking trait anxiety and cognitive biases suggests that high trait anxiety likely increases interference from threat-related distractors like angry faces (Bar-Haim et al., 2007) and emotional, relative to non-emotional, words (Williams et al., 1996). Although associations between anxiety and cognitive interference are well-established, age-related modulation of this purported vulnerability has not been well characterized, including the extent to which adolescence might be a developmental period that is particularly sensitive to the influences from emotionally-salient social distractors such as angry facial expressions.

Just as trait anxiety is seen as a vulnerability factor for clinically-significant anxiety, effortful control is thought to be protective (Derryberry and Reed, 2002). Defined as the ability to inhibit a dominant response to perform a subdominant one, effortful control is thought to play an important role in self-regulation (Rothbart et al., 2004). Developmental studies have consistently linked effortful control with individual differences in a range of self-regulatory behaviors (e.g., Tan et al., 2013) and noted the modulatory influence of effortful control on links between high trait anxiety and cognitive performance impairments (e.g., Derryberry and Reed, 2002; Lonigan and Vasey, 2009). Taken together, it seems likely that individuals with high levels of trait anxiety and low levels of effortful control would show the most interference from distractor stimuli that are thought to signal social threat.

### Summary

Using the Emotional Identification and Dynamic Faces (EIDF) task, the primary objective of the study is to examine the influence of socially-salient emotional and non-emotional distractor stimuli on an important aspect of cognitive control, regulation of interference from task-irrelevant information. The EIDF task asks individuals to focus on a task-relevant color flash that is superimposed on distracting (task-irrelevant) stimuli that vary in social and emotional salience. It includes three types of dynamic distractor stimuli, specifically, (1) *socially-salient but emotionally-neutral* (i.e., faces that change from one identity to another), (2) *socially- and emotionally-salient* (i.e., a face that changes from 0% to 100% emotional), and (3) *non-social/non-emotional* (i.e., a shape that changes in size). The degree to which these different distractor stimuli influence cognitive performance will be indexed by two measures of task performance, accuracy and reaction times on correct trials (correct RT), as well as indices of cognitive interference. Specifically, the degree of interference from socially- and emotionally-salient distractors is calculated using difference scores comparing (1) correct RT on trials that include emotional-social distractors with trails that include neutral-social and non-social/non-emotional distractors and (2) accuracy on trials that include emotional-social distractors as compared to trials with the other distractor types. Notably, the design of the EIDF task allows for investigation of an understudied aspect of “interference” from socially- and emotionally-salient distractors—that there can be conditions whereby social and emotional information might be associated with improved cognitive performance. Because distractor stimuli in the EIDF task are spatially-relevant to the task objective, greater processing of distractors could be reflected in *improved* task performance (i.e., *less* cognitive interference as evidenced by faster and/or more accurate performance).

Thus, consistent with neural and behavioral evidence of heightened social and emotional saliency during adolescence, we expect to find an age × distractor interaction such that relative to adults and children, adolescents will exhibit the fastest correct RT and highest accuracy levels on trials that include non-social/non-emotional distractors, followed by trials with neutral-social distractors, and then trials that include emotional-social distractors. We also expect age-related differences in scores indexing the relative influence of emotional-social distractor stimuli, as compared to neutral-social and non-social/non-emotional distractors. Specifically, we expect a curvilinear developmental pattern whereby relative to children and adults, adolescents show the largest differences in responses to distractor stimuli that are both socially- and emotionally- salient (i.e., emotional facial expressions) as compared to distractors that are only socially-salient (i.e., neutral faces) and, in particular, distractors that have low social and emotional salience (i.e., shapes). Furthermore, because cognitive control systems undergo significant maturational development throughout the adolescent years, we hypothesize that children will exhibit significantly slower correct RTs on trials involving emotional-social distractors than adults. In addition, we expect to find that among emotional-social distractors, angry, and fearful stimuli will show the greatest influence on EIDF task performance at each age, with adolescents showing the greatest effect for angry facial expressions as anger might be particular evocative of adolescents' sensitivity to social threats.

A final, exploratory study goal is to examine the modulatory influence of two affective traits relevant to anxiety disorders, trait anxiety and effortful control, on interference to emotional-social distractors. The present study was designed to elucidate age-related differences in interference to dynamic emotional expressions and therefore underpowered for investigating individual differences; however, as a preliminary step toward understanding individual differences in this aspect of cognitive and affective processing, we explore associations between trait anxiety/effortful control and interference to emotional-social distractors. We predict that (a) high trait anxiety and low effortful control will be associated with greater interference to negative emotional faces, particularly angry facial expressions, (b) that these modulatory influences will be strongest for the adolescent age group, and (c) that regardless of age, trait-anxious individuals with higher levels of effortful control will show less interference than trait-anxious individuals with less effortful control. Significant findings from this exploratory investigation serve as preliminary data for informing future studies of cognitive interference as well as etiological models of anxiety disorders.

## Materials and methods

### Participants

Forty-nine participants (ages 8–52y) were recruited from the community through flyers and other research studies. All were free of neurological, developmental, and psychiatric disorder. Two participants (1 child; 1 adult) were excluded due to extremely low accuracy rates on the EIDF task (percentage correct that is 2*SD* lower than *M*_agegroup_) and one (adolescent) was excluded due to poor task performance (<50% percent correct), resulting in a final sample size of 46. Excluded participants did not significantly differ from the final sample in any socio-demographic characteristics (all *p*'s > 0.34). This final sample included 12 children (8–12y; 7 males), 17 adolescents (13-17y; 7 males) and 17 adults (18–52y; 7 males).

All descriptive data (see Table 1) and results reflect this final sample. An independent *t*-test suggested that the average pubertal development scale score (PDS) was significantly higher in the adolescent than the child group, *t*_(27)_ = −8.47, indicating that the adolescents were not only more advanced in age but also in pubertal maturation (Dorn et al., 2006).

**Table 1:** Socio-demographic characteristics and descriptions of key study variables.

	**Age group**					
	**Children (*****N*** = **12, 7 males)**		**Adolescents (*****N*** = **17, 7 males)**		**Adults (*****N*** = **17,7 males)**	
	***M***	***SD***	***M***	***SD***	***M***	***SD***
Age (years)	9.97	1.00	15.59	1.36	28.80	7.62
Pubertal maturation	1.70	0.59	3.25	0.39	–	–
Effortful Control	3.52	0.39	3.67	0.46	5.18	0.51
Trait Anxiety	42.45	9.95	41.67	8.26	42.62	3.32
**ACCURACY (%)**						
Fearful Face	87	10	92	10	94	7
Angry Face	85	11	93	9	91	9
Sad Face	84	11	93	10	93	8
Happy Face	86	14	94	10	92	8
Non-emotional Face (Ident)	85	16	91	10	92	9
Shape	82	12	89	14	89	9
**REACTION TIME (MS)**						
Fearful Face	1053.95	149.96	933.84	130.83	881.94	102.64
Angry Face	1070.14	161.32	939.81	138.96	896.04	109.15
Sad Face	1057.34	162.62	934.23	120.88	884.90	101.02
Happy Face	1069.12	150.87	946.89	137.24	899.49	104.64
Non-emotional Face (Ident)	1060.43	158.85	936.40	135.96	880.02	91.29
Shape	1049.93	161.43	969.10	151.04	876.84	102.36
**DEGREE OF EMOTIONAL INTERFERENCE (MS)**						
Angry Face RT-Ident RT	−6.48	62.00	−2.56	36.80	1.92	42.94
Fearful Face RT- Ident RT	9.71	56.72	3.41	39.38	16.02	39.95
Sad Face RT- Ident RT	−3.09	67.31	−2.17	48.18	4.88	41.36
Happy Face RT- Ident RT	8.69	51.14	10.50	49.78	19.48	33.15
**DEGREE OF EMOTIONAL INTERFERENCE (% CORRECT)**						
Angry Face ACC-Ident ACC	2	9	10	6	3	6
Fearful Face ACC- Ident ACC	0	10	2	6	0	7
Sad Face ACC- Ident ACC	0	10	2	6	0	6
Happy Face ACC- Ident ACC	1	9	3	6	0	6
**DEGREE OF SOCIAL INTERFERENCE (MS)**						
Angry Face RT-Shape RT	9.01	82.29	−35.35	39.07	5.00	42.42
Fearful Face RT-Shape RT	20.21	61.85	−29.38	45.01	19.20	32.83
Sad Face RT-Shape RT	7.40	55.73	−34.96	50.68	8.06	21.27
Happy Face RT-Shape RT	19.18	80.37	−22.30	46.17	2.26	37.74
**DEGREE OF SOCIAL INTERFERENCE (% CORRECT)**						
Angry Face ACC-Shape ACC	6	8	4	6	5	9
Fearful Face ACC-Shape ACC	3	9	4	7	2	9
Sad Face ACC-Shape ACC	2	10	4	9	4	8
Happy Face ACC-Shape ACC	4	8	5	7	2	8

*Child and adolescent levels of trait anxiety were assessed using Spielberg's State and Trait Anxiety Inventory for Children (STAI-C); Adult trait anxiety was assessed using the State-Trait Anxiety Inventory (STAI). Youth's self-perception of their pubertal status was assessed using the Pubertal Development Scale (PDS). Attentional control was assessed using the Early Adolescent Temperament Questionnaire (EATQ) in children and adolescents and the Adult Temperament Questionnaire (ATQ) in adults. Scores indexing individual differences in trait anxiety and temperamental attentional control were standardized using z-scores in analyses*.

### Procedures

All study procedures were approved by the university's Institutional Review Board. Parents gave written informed consent for their child's participation in the study; youth also gave written informed assent in accordance with the Declaration of Helsinki. At the laboratory, participants were screened for presence of psychopathology using the Child, Adolescent, or Adult Symptom Inventory, which is a well-validated checklist that provides a broad measure of DSM-based diagnoses (DSM-IV; American Psychiatric Association, 2000). Specifically, parents of youth ≥12 years completed the Adolescent Symptom Inventory-4 (ASI−4; Gadow and Sprafkin, 1998); parents of youth <12 years completed the Child Symptom Inventory-4 (CSI– 4; Gadow and Sprafkin, 1998). Adult participants completed the Adult Self-Report Inventory-4 (ASRI−4; Gadow et al., 2004). Following the EIDF task, participants completed measures of pubertal status, trait anxiety, and effortful control. Parents gave written informed consent for children's participation.

#### Emotional identification and dynamic faces (EIDf) task

**Figure 1:**
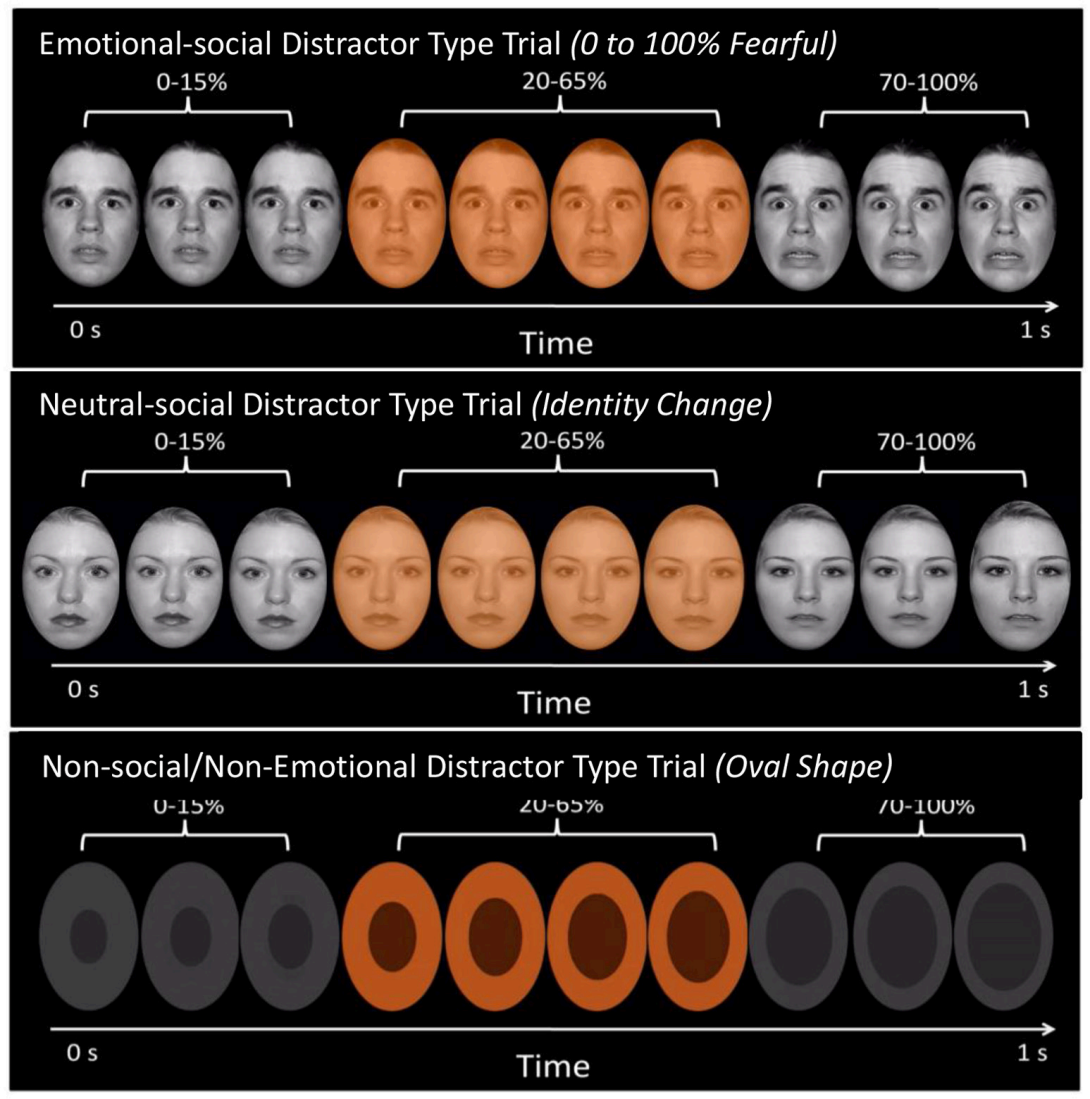
Depiction of EIDI task. Each of the three distractor type conditions are shown above. First, trials that included emotionally- and socially salient distractors, i.e., emotional facial expressions that changed from 0% (neutral) to 100% emotional. These emotional-social traits included four type of emotional expressions, angry, fearful, sad, and happy. Second trials that included neutral, but socially-salient distractors, i.e., neutral facial expressions that changed identity. Last, trials that included non-social/non-emotional distractors i.e., oval shapes that increased in size. In every trial, a color flash would occur midway through the 1-second clip. The color flash is superimposed on the dynamically changing face or oval stimuli. Individuals were asked to respond, by pressing one of three buttons, to whether the color flash was orange, blue, or yellow as quickly and accurately as possible.

Eligible participants completed a practice task, which was a shortened version of the EIDF task with different stimuli from the actual task, during which the experimenter presented instructions regarding performing the task and which keyboard button to press. Following the 5-min practice session, participants completed the 15-min EIDF task (see Almeida et al., 2011; Surguladze et al., 2012). As shown in Figure 1, the EIDF comprised of trials with emotional-social (emotional facial expressions that changed from 0% angry/fearful/sad/ happy), neutral-social (dynamic faces that changed from one identity to another) and non-social/non-emotional (dynamic ovals that increased in size) distractors. In trials with emotional-social distractors, a semi-transparent color filter was superimposed on gray-scale faces that were morphed from neutral (0% emotion) to fully (100%) emotional in 5% increments using the Winmorph software program. Trials with neutral-social distractors were similarly designed, except that the color filter was superimposed on gray-scale faces that were morphed from one identity to another, also in 5% increments. Trials with non-social/non-emotional distractors comprised of gray-scale shapes (that were superimposed with a color filter as they increased in size. Facial stimuli were generated from the NimStim stimuli set (Tottenham et al., 2009) and included 12 individual faces (2 male and 2 female from African-American, Asian, and Caucasian ethnic backgrounds). The non-social/non-emotional distractor stimuli consisted of a dark oval superimposed on a light-gray oval with similar structural characteristics to facial stimuli and changed in size in a manner that approximated the movement of facial changes.

Participants had to identify the color of the filter as quickly and accurately as possible by pressing one of three keyboard buttons to indicate if the color flash was orange, blue, or yellow by using one of three fingers on his/her dominant hand. The flash appeared during the middle portion of the 1-s movie of the morphing face/shape, between ~200 and 600 ms, when emotional expressions and identity changes were between 25 and 50%. Participants were shown four blocks for each of the four emotional-social stimuli (12 stimuli per block; fearful, angry, sad, happy), and six control blocks (6 stimuli per block), so that a single emotion block was not repeated sequentially (see Hafeman et al., 2014).

#### Emotion labeling task

In order to identify a potential confound for interpreting age-related differences in EIDF task performance on trials involving emotional-social distractor stimuli, we used the Emotional Labeling task to investigate possible age differences in participants' ability to identify dynamic angry, fearful, sad, and happy emotional facial expressions. This task included dynamic emotional-social stimuli (i.e., faces the morphed from 0 to 100% emotional) that were identical in design to those used in the EIDF task. During this task, participants used one of four fingers on one hand to press a button indicating whether a dynamically-emerging facial expression should be labeled as “angry,” “fearful,” “sad,” or “happy.” The task consisted of 2 blocks of 40 trials; each block included 10 trials for each emotional condition.

### Measures

#### Trait anxiety

The 20-item Trait scale from the State–Trait Anxiety Inventory for Children (STAI-C; Spielberger, 1973) was used to assess youth's trait anxiety or general predisposition to experience anxiety. Adult participants completed the 20-item inventory (STAI; Spielberger et al., 1977). Both versions used a 4-point scale to rate whether items such as “I lack self-confidence” were not at all, somewhat, moderately, or very true and showed adequate reliability (α = 0.78 for the STAI-C and α = 0.76 for the STAI). Ratings for each item are summed, with larger values reflecting higher levels of trait anxiety.

#### Effortful control

The Early Adolescent Temperament Questionnaire (EATQ-R; Ellis and Rothbart, 2001) assessed youth's effortful control using the Effortful Control factor score from the validated 65-item short form (Muris and Meesters, 2009), which had adequate reliability in the present sample (α = 0.74). The adult version of the EATQ, the 77-item short from of the Adult Temperament Questionnaire (ATQ; Evans and Rothbart, 2007) was used with adults and also showed adequate reliability in the present sample (α = 0.77). The Effortful Control factor score was calculated by averaging ratings on items from the attentional, inhibitory, and activation control scales; Factor scores can range from 1 to 5, with larger values reflecting higher levels of effortful control.

#### Pubertal status

Youth's self-perceived pubertal status was assessed via the widely-used Pubertal Development Scale (PDS; Petersen et al., 1988), which showed adequate reliability (α = 0.94 for females, α = 0.93 for males) in the present sample. Boys rated facial hair and voice deepening while girls rated breast development and menarcheal status. Both sexes also rated their maturational status on three markers of pubertal development (pubic hair growth, skin changes, and growth spurt, on a four-point scale ranging from (1) not yet present to (4) fully developed. The sets of items form scales consisting of five items, which are coded on a 4-level ordinal response scale. An average pubertal development score was computed by summing across the five items, and then divided by five, to preserve the original (1–4) metric.

#### Statistical analysis

Outlying reaction time (RT) data points <100 or >3,000 ms were excluded, comprising <1% of trials. Because preliminary analyses suggested no associations between sociodemographic variables and EIDF performance measures, covariates such as child sex and parental education are not further considered [all *p's* >0.30, with exception of accuracy on fear trials (*p* = 0.08)]. Table 2 presents bivariate correlations between key study variables.

**Table 2:** Bi-variate correlations of key study variables.

**Variable**	**1**	**2**	**3**	**4**	**5**	**6**	**7**	**8**	**9**	**10**	**11**	**12**	**13**	**14**	**15**	**16**	**–**	**18**	**19**	**20**	**21**	**22**
1. FearCorRT	–	–	–	–	–	–	–	–	–	–	–	–	–	–	–	–	–	–	–	–	–	–
2. AngCorRT	0.95^**^	–	–	–	–	–	–	–	–	–	–	–	–	–	–	–	–	–	–	–	–	–
3. Sad_CorRT	0.92^**^	0.94^**^	–	–	–	–	–	–	–	–	–	–	–	–	–	–	–	–	–	–	–	–
4. HapCorRT	0.94^**^	0.93^**^	0.95^**^	–	–	–	–	–	–	–	–	–	–	–	–	–	–	–	–	–	–	–
5. IdentCorRT	0.95^**^	0.96^**^	0.94^**^	0.95^**^	–	–	–	–	–	–	–	–	–	–	–	–	–	–	–	–	–	–
6. ShapeCorRT	0.93^**^	0.94^**^	0.95^**^	0.93^**^	0.95^**^	–	–	–	–	–	–	–	–	–	–	–	–	–	–	–	–	–
7. FearAcc	0.07	0.10	0.11	0.06	0.09	0.18	–	–	–	–	–	–	–	–	–	–	–	–	–	–	–	–
8. AngAcc	0.03	0.04	0.07	0.06	0.06	0.16	0.82^**^	–	–	–	–	–	–	–	–	–	–	–	–	–	–	–
9. SadAcc	−0.01	0.01	0.04	0.03	0.06	0.11	0.79^**^	0.81^**^	–	–	–	–	–	–	–	–	–	–	–	–	–	–
10. HapAcc	0.16	0.19	0.18	0.10	0.16	0.27	0.81^**^	0.81^**^	0.79^**^	–	–	–	–	–	–	–	–	–	–	–	–	–
11. IdentAcc	0.11	0.14	0.10	0.06	0.11	0.19	0.80^**^	0.77^**^	0.78^**^	0.82^**^	–	–	–	–	–	–	–	–	–	–	–	–
12. ShapeAcc	0.20	0.20	0.19	0.15	0.17	0.26	0.83^**^	0.78^**^	0.74^**^	0.82^**^	0.80^**^	–	–	–	–	–	–	–	–	–	–	–
13. FearShapeDiff (Fear - Shape)	0.02	−0.13	−0.23	−0.12	−0.18	−0.36^*^	−0.29^*^	−0.37^*^	−0.33^*^	−0.32^*^	−0.24	−0.20	–	–	–	–	–	–	–	–	–	–
14. AngShapeDiff (Ang–Shape)	0.05	0.13	−0.05	−0.02	−0.03	−0.21	−0.24	−0.35^*^	−0.31^*^	−0.22	−0.16	−0.17	0.68^**^	–	–	–	–	–	–	–	–	–
15. SadShapeDiff (Sad–Shape)	−0.20	−0.20	−0.05	−0.14	−0.24	−0.36^*^	−0.23	−0.30^*^	−0.26	−0.32^*^	−0.32^*^	−0.25	0.46^**^	0.50^**^	–	–	–	–	–	–	–	–
16. HapShapeDiff (Hap–Shape)	−0.08	−0.15	−0.13	0.07	−0.12	−0.32^*^	−0.30^*^	−0.28	−0.24	−0.44^**^	−0.37^*^	−0.30^*^	0.65^**^	0.49^**^	0.61^**^	–	–	–	–	–	–	–
17. FearIdentDiff (Fear–Identity)	0.10	−0.06	−0.10	−0.09	−0.22	−0.14	−0.07	−0.11	−0.21	−0.02	0.00	0.08	0.61^**^	0.23	0.14	0.13	–	–	–	–	–	–
18. AngIdentDiff (Ang–Identity)	0.14	0.26	0.13	0.03	−0.03	0.08	0.03	−0.05	−0.15	0.14	0.12	0.14	0.13	0.53^**^	0.13	−0.13	0.52^**^	–	–	–	–	–
19. SadIdentDiff (Sad–Identity)	−0.12	−0.09	0.12	−0.08	−0.23	−0.06	0.05	0.03	−0.06	0.04	−0.04	0.06	−0.14	−0.08	0.55^**^	−0.03	0.35^*^	0.46^**^	–	–	–	–
20. HapIdentDiff (Hap–Identity)	−0.02	−0.09	0.02	0.15	−0.16	−0.09	−0.09	−0.01	−0.10	−0.18	−0.18	−0.05	0.19	0.01	0.35^*^	0.61^**^	0.43^**^	0.21	0.51^**^	–	–	–
21. Trait Anxiety	−0.06	−0.03	−0.13	−0.19	−0.13	−0.16	−0.11	−0.10	−0.25	−0.10	0.01	−0.05	0.27	0.35^*^	0.11	−0.06	0.24	0.31^*^	0.02	−0.18	–	–
22. Effortful Control	−0.36^*^	−0.38^**^	−0.35^*^	0.35^*^	−0.38^**^	−0.38^**^	0.33^*^	0.17	0.34^*^	0.20	0.17	0.23	0.13	0.04	0.18	0.14	0.11	−0.03	0.11	0.13	−0.17	–

* *indicates p < 0.05*;

** *indicates p < 0.01*.

#### Indexing task performance

The influence of socially- and emotionally-salient distractors on cognitive performance in the EIDF task is assessed using accuracy (percentage correct) and response times on correct trials (correct RTs). Specifically, mixed ANOVA models were used, with age (Child, Adolescent, Adult) as a between-subject variable and distractor type (neutral-social, fearful-social, angry-social, sad-social, happy-social, and non-social/non-emotional) as within-subject variable. A-priori hypotheses concerning age-related differences on trials with different types of distractors (i.e., emotional-social vs. neutral-social vs. non-emotional/non-social) were tested using contrasts. All *post-hoc* age group comparisons were conducted using Tukey's HSD Adjustment for multiple comparisons.

#### EmoShapeDiff scores indexing interference from emotionally- and socially-salient distractors: EmoShapeDiffrt and EmoShapeACC

Participants' degree of interference that is associated to with the social and/or emotional saliency of distractor stimuli is measured using two sets of differences scores. The first set of difference scores, herein referred to as *EmoShapeDiff* scores, were created by subtracting the average RT of correct, non-social/non-emotional (shape) trials from the average RT of correct, emotional-social trials (e.g., Angry Morph—Shape Morph). Positive values on EmoShapeDiffRT scores are indicative of greater interference from distractor stimuli with a high degree of social and emotional salience, relative to shape distractors with putatively no social or emotional salience. Negative values on these difference scores are indicative of less interference, meaning that a person is faster to correctly respond to trials with an emotional facial expression distractor than trials with a shape distractor. The same approach was used to create difference scores associated with the effect of emotionally- and socially-salient distractors on accuracy levels. Negative values on the EmoShapeDiffACC scores are indicative of greater interference, meaning that a person is less accurate on trials with an emotional face distractor than trials with a non-emotional/non-social shape distractor.

#### EmoIdentDiff scores indexing interference specific to the emotional salience of social distractors: EmoIdentDiffRT and EmoIdentDiffACC

Next, a second set of differences scores, herein referred to as *EmoIdentDiffRT and EmoIdentDiffACC*, were created in order to differentiate between the modulatory influence of face distractors that is general to social stimuli (i.e., neutral face that changes identity or neutral-social distractors) vs. specific to *emotionally-salient* social stimuli (i.e., face that changes from 0 to 100% emotional or emotional-social distractors). Specifically, EmoIdentDiffRT scores were created by subtracting the average RT of correct, neutral-social trials from the average RT of correct, emotional-social trials (e.g., Angry Morph—Identity Morph). Likewise, EmoIdentDiffACC scores were created by subtracting the average accuracy level of neutral-social trials from emotional-social trials. Positive values on EmoIdentDiffRT scores and negative values on EmoIdentDiffACC scores are indicative of interference effects that are *specific to the emotional saliency* of socially-relevant distractors. In contrast, negative values on reaction time difference scores and positive values on accuracy difference scores are indicative of possible faciliatory effects that are specific to emotional distractors.

After creating difference scores indexing cognitive interference, mixed ANOVA models, with age (Child, Adolescent, Adult) as a between-subject variable and difference score type (fearful-social, angry-social, sad-social, happy-social) as within-subject variable, were used to test hypotheses regarding age-related differences between the socially- and emotionally-salient distractors and (1) non-social/non-emotional as well as (2) non-social/non-emotional distractors. All *post-hoc* age group comparisons were conducted using Tukey's HSD Adjustment for multiple comparisons.

Finally, regression models were used to explore individual differences in cognitive interference due to the social and emotional saliency of distractors (i.e., EmoShapeDiff scores). Because adults' trait anxiety and effortful control were assessed using different measures than youth, we used standardized (*z*-scores) to index variability across age groups. Participant age (in years), standardized trait anxiety and effortful control *z*-scores, and a trait anxiety × effortful control interaction term, served as predictors.

## Results

### Preliminary analysis: age-related differences in the emotion labeling task

Two one-way ANOVA models revealed no age-related effects on emotion recognition ability, as indexed by accuracy, all *p's* > *0*.470, and response times on correct trials, *p's* > *0*.324 from the Emotion Labeling task. This preliminary analysis suggests that if evident, age-related differences in EIDF task performance are likely not due to age-related differences in *recognition* of specific emotional expressions.

### Age-related differences in accuracy and reaction time

**Figure 2:**
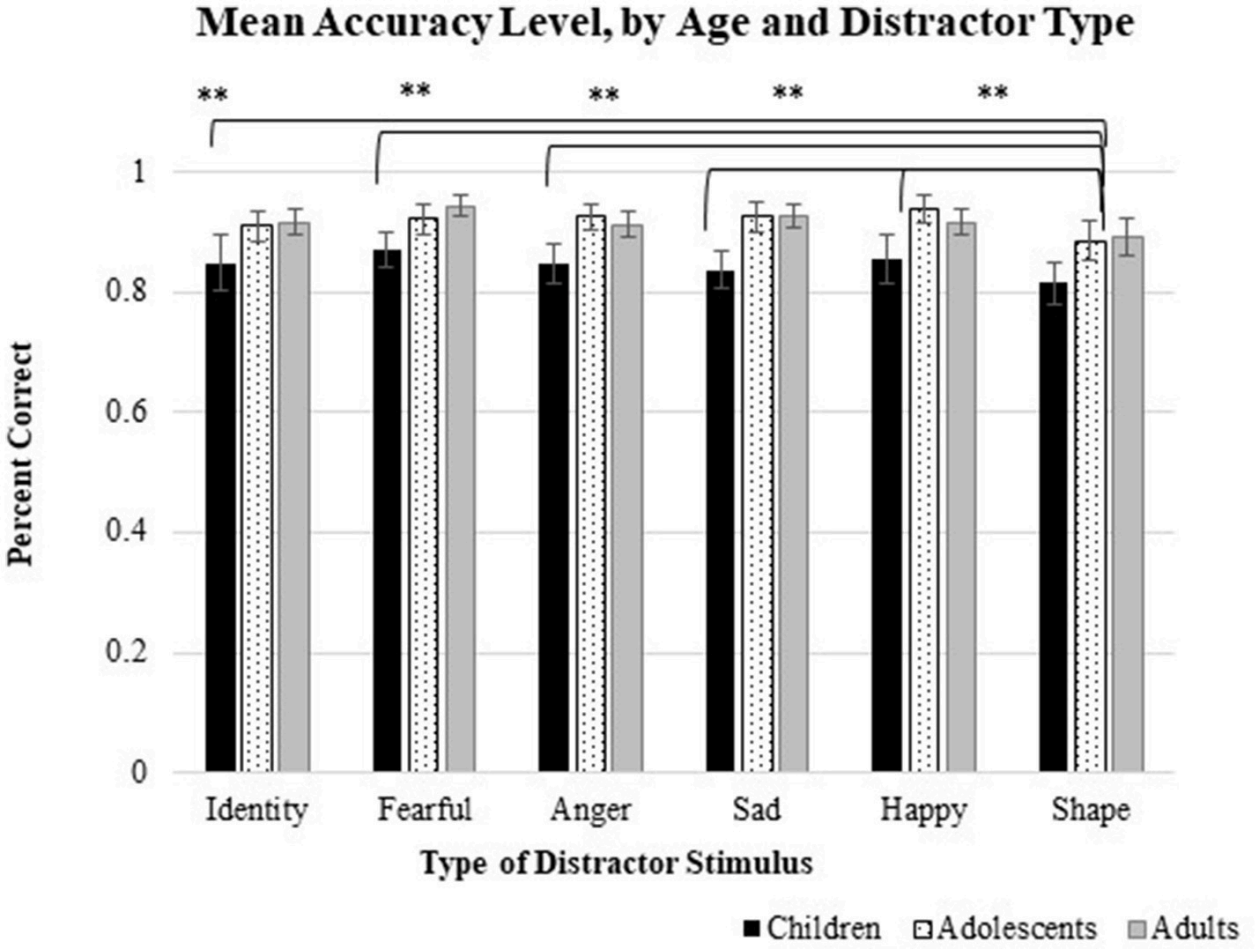
Modulatory influence of distractor type on mean accuracy. As shown above, individuals showed higher accuracy levels on trials that included distractor stimuli with high social salience (i.e., trials where faces changed from 0 to 100% neutral and changed identity), relative to trials with non-social/non-emotional distractors (i.e., trials where ovals increased in size). There were no effects of *specific emotion* type on accuracy level and no significant age × distractor type interaction effect, suggesting that accuracy levels did not differ as a function of a specific negative (fearful, angry, sad) or positive (happy) facial expression. ^**^indicates *p* < 0.01.

Results from mixed (age group × distractor type) ANOVA models revealed a main within-subjects effect of distractor type on accuracy, *F*_(5, 39)_ = 4.57, *p* = 0.001, η^2^ = 0.10. As seen in Figure 2, Tukey HSD-adjusted *post-hoc* tests indicated that participants were more accurate on trials that included socially-salient distractor stimuli (both emotional-social and neutral-social distractor types) than on trials that included distractors with low social and emotional saliency (non-social/non-emotional distractor). Additionally, there were no significant differences between trials with angry or fearful distractors, relative to trials that included neutral, sad, or happy distractors. There were no significant age-related effects on accuracy, *F*(_2, 43_) = 2.53, *p* = 0.09, η^2^ = 0.11 nor was there a significant age Group × distractor type interaction effect, *F*_(10, 80)_ = 0.578, *p* = 0.83, η^2^ = 0.03.

Results from a second mixed (age group × distractor type) ANOVA model examining correct reaction times (correct RTs) revealed a main effect of age, *F*_(2, 43)_ = 6.65, *p* = 0.003, η^2^ = 0.24. Tukey HSD-adjusted *post-hoc* tests indicated that children (*M* = 1,060, *SE* = 36.64) were significantly slower on correct trials than adults (*M* = 886.5, *SE* = 30.78), *M*_*Difference*_ = 173.6, *SE*_*Difference*_ = 47.86, *p* = 0.002) and adolescents (*M* = 9 43.4, *SE* = 30.78), *M*_*Difference*_ = 116.8, *SE*_*Difference*_ = 47.86, *p* = 0.049. Adolescents and adults did not show a significant difference on correct RTs, *M*_*Difference*_ = 56.86, *SE*_*Difference*_ = 43.53, *p* = 0.400. There was no significant distractor type, *F*_(5, 39)_ = 1.41, *p* = 0.22, η^2^ = 0.03, age group × distractor type interaction effect, *F*_(10, 80)_ = 1.45, *p* = 0.16, η^2^ = 0.06^1^.

### Age-related differences in interference from emotional-social vs. non-social/non-emotional distractors: EmoShapeDiffRT and EmoShapeDiffACC scores

**Figure 3:**
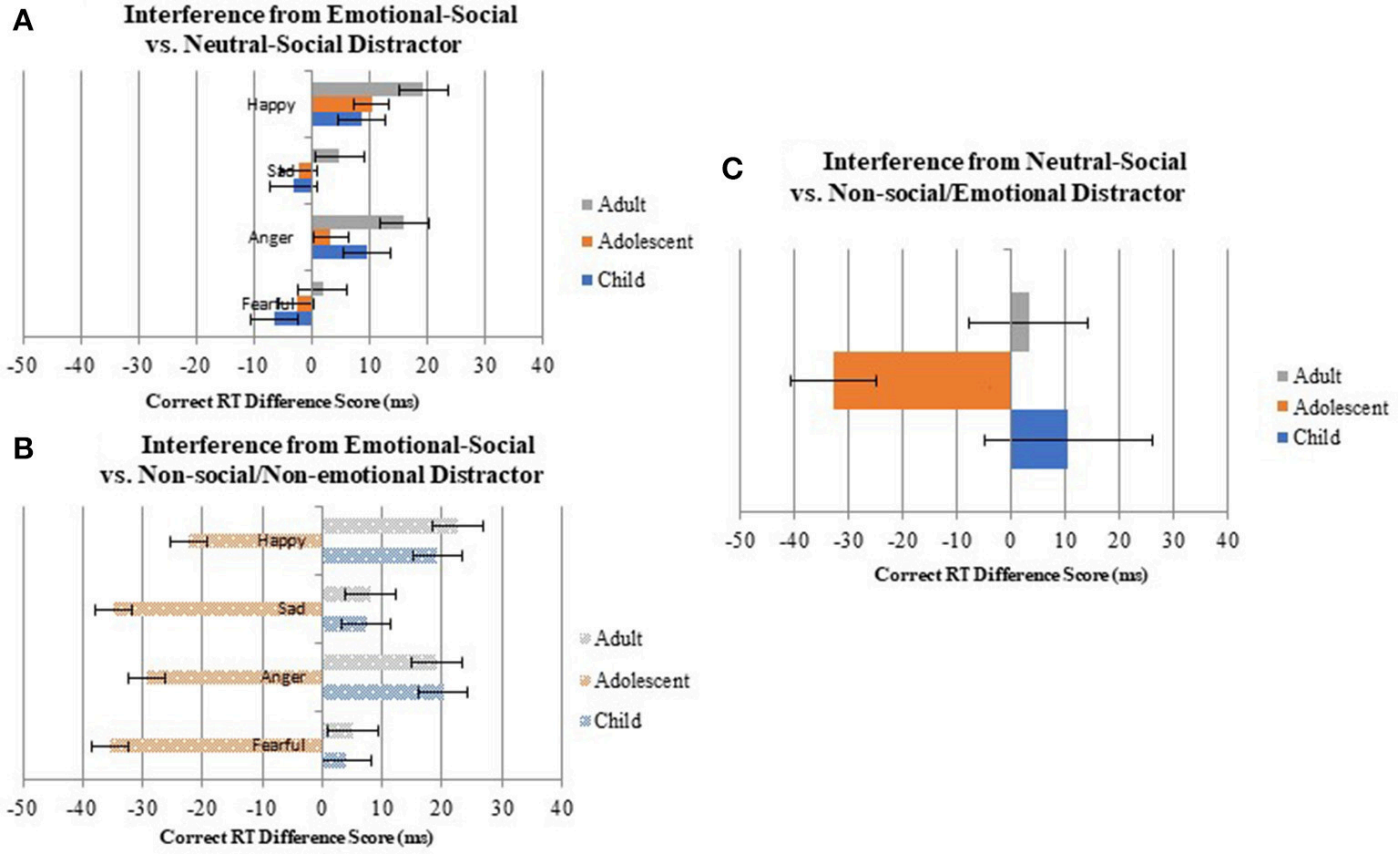
Differing scores indexing interference from emotional-social distractor stimuli, relative to **(A)** neutral-social and **(B)** non-social/non-emotional distractors. and **(C)** neutral-social distractor stimuli, relative to non-social/non-emotional distractors. **(A)** Depicts EmoIdenDiff different scores indexing the relative influence of emotionally-salient social distractors (i.e., faces that change from neutral to 100% emotional) on correct reaction times, relative to neutral social distractors (i.e., faces that change identity). **(B)** Depicts EmoShapeDiff difference scores indexing the relative influence of emotionally-salient social distractors as compared to non-social/non-emotional distractors (i.e., ovals that increase in size). As seen in **(B)**, in contrast to adults and children who are slower during correct trials that involve emotional-social distractors (relative to shape distractors), adolescents are faster on trials that include these distractors. However, as seen in **(A)**, this age-related pattern is not observed when comparing correct responses from trials without emotional-social-distractors to trials with neutral-social distractors, suggesting that the “advantage” in reaction time that adolescents show in emotional-distractor trials is likely due to the social salience of faces vs. shapes. **(C)** Shows age-related differences in IdentShapeDiff difference scores indexing the relative influence of neutral, but socially-salient, distractors (i.e., neutral faces that change identity) on correct reaction times, relative to non-social/non-emotional distractors (i.e., shapes that increase in size). In contrast to adults and children, who are slower during correct trials that involve neutral face distractors, adolescents are faster on these trials, as compared to non-social/non-emotional shape distractors.

Results from a mixed 3 (age group) × 4 (EmoShape difference score type) ANOVA model indicated that interference associated with social-emotional distractors, as indexed by reaction time, significantly differed by age. Specifically, there was a significant main effect of age, *F*_(2, 43)_ = 6.74, *p* = 0.003, η^2^ = 0.24. Tukey HSD-adjusted *post-hoc* tests indicated that children (*M* = 12.70, *SE* = 10.18) and adults (*M* = 13.75, *SE* = 9.47) showed a similar degree of interference, *M*_*Difference*_ = −1.05, *SE*_*Difference*_ = 14.73, *p* = 1.00. However, adults showed more interference from social-emotional distractors than adolescents (*M* = −30.752), *M*_*Difference*_ = 44.25, *SE*_*Difference*_ = 13.40, *p* = 0.006. Likewise, children also showed more interference from social-emotional distractors than adolescents, *M*_*Difference*_ = 43.20, *SE*_*Difference*_ = 14.73, *p* = 0.016. That is, children and adults exhibited positive EmoShapeDiffRT scores, indicating that they were slower to correctly respond on trials with non-social/non-emotional (shape) distractors than on trials with emotional-social distractors (Figure 3). There was no significant effect of EmoShape difference score type, *F*_(3, 84)_ = 1.90, *p* = 0.133, η^2^ = 0.04 or an age group × difference score type interaction, *F*_(6, 84)_ = 0.07, *p* = 0.999, η^2^ = 0.003. These results suggest that “interference” due to the presence emotional-social distractors, that is, changes in correct RTs associated with the presence of emotional-social distractors, did not differ across specific (angry, fearful, sad, happy) emotions. In contrast, results from a second mixed ANOVA model of interference as indexed by accuracy level did not significantly differ by age, *F*_(2, 43)_ = 0.07, *p* = 0.929, η^2^ = 0.00 or difference score type, *F*_(3, 84)_ = 1.21, *p* = 0.309, η^2^ = 0.03. Similarly, there was no significant age group × EmoShape difference score type interaction, *F*_(6, 84)_ = 1.15, *p* = 0.343, η^2^ = 0.01 on accuracy.

### Age-related differences in interference from emotional-social vs. neutral-social distractors: EmoIdentDiffRT and EmoIdentDiffACC scores

Mixed 3 (age group) × 4 (EmoIdent difference score type) ANOVA models of EmoIdentDiffRT and EmoIdentDiffACC examined whether emotional salience of faces is associated with cognitive interference. Results revealed no significant main within-subjects effects of EmoIdent difference score type, *F*_(3, 84)_ = 1.90, *p* = 0.133, η^2^ = 0.04, or between-subjects effects of age, *F*_(2, 43)_ = 0.30, *p* = 0.742, η^2^ = 0.01 for reaction time. In addition, there was no significant age group × EmoIdent difference score type interaction effect, *F*_(6, 84)_ = 0.07, *p* = 0.999, η^2^ = 0.03. Results from the mixed ANOVA examining accuracy level difference scores (EmoIdentDiffACC) indicated no significant age, *F*_(2, 43)_ = 0.30, *p* = 0.742, η^2^ = 0.01, or EmoIdent difference score type, *F*_(3, 84)_ = 1.90, *p* = 0.133, η^2^ = 0.04, effects. There was also no significant age group × difference score type interaction, *F*_(6, 84)_ = 0.07, *p* = 0.999, η^2^ = 0.003.

A follow-up one way ANOVA model examining age effects on correct RTs on trials with neutral-social and non-social/non-emotional distractors revealed a similar pattern. A difference score was first created by subtracting average correct RTs from non-social/non-emotional distractor trials from neutral-social distractor trials (Identity—Shape correct RTs). An age effect was observed, *F*_(2, 43)_ = 4.45*, p* = 0.017, η^2^ = 0.17 (see Figure 3), indicating that adolescents were significantly faster than children and adults on correct trials with a socially-salient distractor than trials with a non-social/non-emotional distractor (i.e., oval shape), *M*_*DifferenceAdolescChild*_ = −43.28, *SE*_*DifferenceAdolescChild*_ = 16.31, *p* = 0.011; *M*_*DifferenceAdolescAdult*_ = −35.97, *SE*_*DifferenceAdult*_ = 14.84, *p* = 0.020.

### Individual differences in emotional and social interference

**Figure 4:**
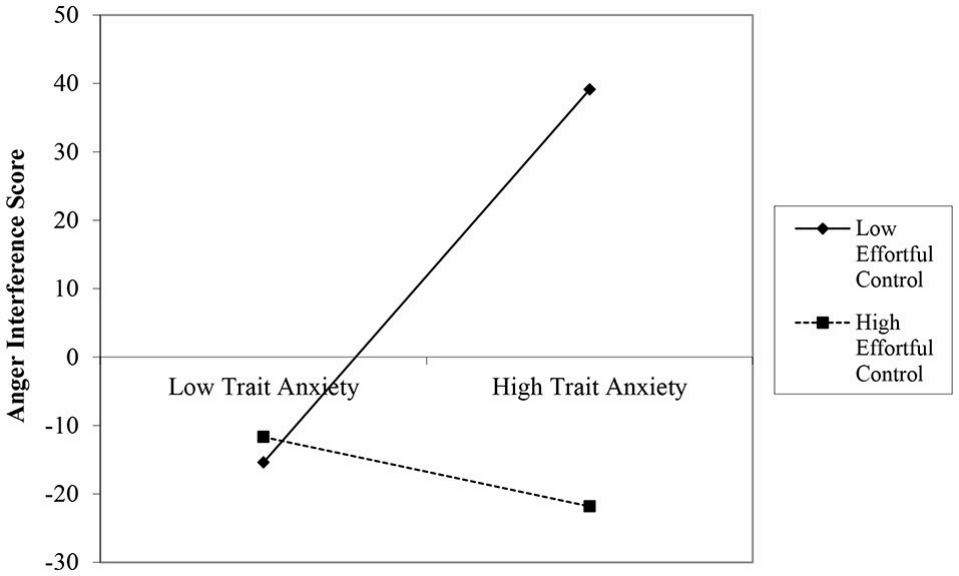
Relations between Trait Anxiety × Effortful Control and degree of emotional interference to angry distractors. As shown in Figure 1, individuals with high levels of effortful control (dotted line; for illustrative purposes herein defined as 1 *SD* above the sample mean) showed a similar degree of interference to angry faces across low and high levels of trait anxiety (depicted here at ± 1*SD* the sample mean). In contrast, the degree to which individuals with low levels of temperamental effortful control show interference to angry faces (solid line; defined as 1 *SD* below the sample mean) is associated with their levels of trait anxiety. Specifically, individuals with low levels of effortful control and trait anxiety show significantly less interference than individuals with low levels of effortful control but high levels of trait anxiety, *t* = 2.86, *p* = 0.007 whereas individuals with high levels of trait anxiety and high levels of effortful control show a similar degree of interference as individuals with low levels of trait anxiety. Age is not significantly associated with relations between affective traits and emotional interference. Standardized scores of temperamental effortful control and trait anxiety were used as predictors of angry interference scores.

Given the small sample size, four exploratory hierarchical regression were used to explain the influence of trait anxiety and temperamental effortful control on interference from distractor stimuli with high social and emotional salience. Regression models were significant only for interference specific to angry emotional faces, such that trait anxiety and effortful control accounted for significant variance in interference, *F*_(4, 39)_ = 3.64, *p* = 0.013, *R*^2^ = 0.27, relative to non-social distractors. Specifically, the interaction of trait anxiety × effortful control predicted degree of interference from dynamically-emerging angry faces, ß = −0.31, *t* = −2.19, *p* = 0.035. Following guidelines outlined by Aiken and West (1991), this interaction was probed by plotting interference to angry distractors at high (+1*SD*) and low (−1*SD*) levels of trait anxiety and effortful control (Figure 4). Tests of simple slopes (Dawson, 2014) indicated that interference differed between high and low trait anxious individuals with low levels of effortful control, *t* = 2.86*, p* = 0.007. Interference does *not* differ between high and low anxious individuals with *high* effortful control, *t* = −0.479*, p* = 0.634. Trait anxiety and effortful control were not associated interference to other emotional distractors (all *p's* ≥ 0.31, see Supplementary Table 1). Finally, regression models investigating cognitive interference in terms of differences in accuracy levels between emotional and shape trials (i.e., EmoShapeDiffRT) were not significant, all *p's* > 0.30.

## Discussion

Using the EIDF task, this study examined ages-related differences in the modulatory influence of distractors that vary in social and emotional significance, focusing on the degree to which cognitive interference is specific to emotionally-salient task-irrelevant information, in the context of distractors with high and low social salience. As expected, children were significantly slower to respond correctly than adults but they were not less accurate. With regards to cognitive interference on trials with and without emotionally- and socially-salient distractors, we found unexpected results suggesting that in contrast to children and adults, adolescents showed *less* interference on trials with social distractors than on trials with non-social ones. Finally, exploratory analyses examining individual differences in social and emotional interference suggest that the association between trait anxiety and interference to angry distractors seems to be moderated by effortful control across age groups. Although exploratory, these findings are consistent with research suggesting that regardless of age, high anxious individuals with low levels of effortful control may be at particular risk for developing anxiety disorders, in part, because they have difficulties inhibiting/shifting attention away from threat-related information (e.g., Bar-Haim et al., 2007).

Our age-related developmental analysis of cognitive interference, as indexed by difference scores, suggests that under certain conditions, adolescents exhibit increased levels of task engagement—for example, orienting to task demands—that might also facilitate their performance on tasks that require regulation of task-irrelevant information. This finding could suggest that, adolescents exhibit reduced cognitive interference, as indexed by how quickly they could accurately react to task-relevant information that was presented alongside task-irrelevant information, in part because of increased motivation. That is, adolescents may have found faces to be more salient possibly leading to increased arousal and faster response times. Because this behavioral task does not differentiate between increased arousal/motivation and decreased interference/increased regulation, future studies should examine this possibility. In addition, there were no age differences in cognitive interference as indexed by overall accuracy levels suggesting that the influence of socially-salient emotional stimuli is more readily observed when comparing *speed* of correct responses. This could be attributed to a ceiling effect, that is, little variability in the (high) accuracy rates exhibited by all participants on this task.

Two factors are important for understanding this hypothesized, but seemingly counterintuitive, finding. First, adolescence is characterized by heightened saliency of emotional and social information (e.g., Nelson et al., 2005; Somerville et al., 2011). Second, in contrast to other tasks used to assess emotional interference, task-relevant information is superimposed onto distractors in the EIDF task, such that when a person focuses attention on the socially- and emotionally-salient distractor, he/she focuses attention in the same spatial location as the target color flash. Our results also indicate that this effect is *generally* associated with social salience, rather than *specifically* associated with the emotional salience of social stimuli. Taken together, one possible explanation is that adolescents' orientation to socially-salient stimuli could increase their processing of target stimuli information on trials that included faces relative to non-emotional shapes as distractors. In short, even when this information is task-irrelevant, adolescents show a particular increase in task engagement when processing socially-salient information that increases cognitive resources that help them quickly process socially-salient emotional information. We saw this possible “faciliatory” effect in adolescents' correct reaction times, not in overall accuracy levels, suggesting that adolescents were able to more efficiently (i.e., quickly) attend to task-relevant information to maintain sufficient accuracy during the task. This is consistent with emerging evidence of a mid-adolescent increase in activation of neural regions supporting cognitive control (e.g., Crone et al., 2006; Geier et al., 2010), especially when task contexts include socially- and/or affectively-salient stimuli. The merits of this explanation require systematic investigation and replication in future studies.

Nonetheless, there is some preliminary support for interpreting the age-related findings observed in this study—specifically, that relative to adults and children, adolescents show decreased interference in some contexts—as evidence that salient “distractors” can be linked with improved task performance. For example, recent work has suggested that another cognitive control process, conflict monitoring is enhanced by emotional information when the target, and not just the distractor, is emotional (Kanske and Kotz, 2010, 2011). This interpretation is also consistent with theory that adolescents flexibly modulate cognitive control systems depending on the motivational salience (e.g., emotional/social relevance) of the context (Crone and Dahl, 2012), an aspect of cognitive control that might be especially beneficial for learning to read social cues while navigating new and social contexts (e.g., school dances, first dates). It is surprising that few emotion-specific effects emerged. However, the vast majority of studies documenting the particular saliency of negative emotional facial expressions (i.e., anger, fearfulness) relied on static images. The EIDF task included dynamic faces, which may have increased the saliency across emotional expressions, reducing the extent to which individuals show preferential attention to angry faces, relative to non-emotional shapes.

Although future work is needed to identify the cognitive processes underlying adolescents' flexible regulation of interference from task-irrelevant stimuli, including the possibility that they show improved regulation of interference from social vs. non-social stimuli in the EIDF task, our findings highlight the need to understand how variations in task demands and saliency of task-irrelevant distractor stimuli influence different subprocesses of cognitive control at different developmental periods. This is especially important for identifying the neurocognitive mechanisms that underlie elevations in individuals' ability to regulate social cues that are thought to increase for anxiety disorders.

We also explored individual differences in interference from socially- and emotionally-salient distractors, focusing on affective traits that have been implicated in anxiety symptoms and disorders. To better control for Type 2 error, we focused on associations between these affective traits and emotional-social distractors (i.e., EmoShapeDiff scores) because of the particular influence that negative social cues have on the alterations in cognitive control that have been linked with anxiety disorders. In this small sample, we found that effortful control modulates interference to dynamic facial expressions of anger, such that trait-anxious individuals show improved interference control when they also have high effortful control. However, given that we did not conduct prospective power analyses for these exploratory analyses, low power likely limited our ability to detect the modulatory influence of trait anxiety and effortful control for sad, fearful, or happy distractors. Although these exploratory analyses were designed to provide preliminary data regarding individual differences in EIDF performance, our findings are consistent with a body of research suggesting that risk conferred by high trait anxiety may be mitigated by executive control processes (e.g., Derryberry and Reed, 2002). It therefore may be especially important to develop cognitive remediation techniques that for individuals with low effortful control. We had expected that this aspect of cognitive control would be particularly relevant during adolescence but findings instead suggest that the trait anxiety and effortful control modulate interference to angry faces (relative to non-social stimuli) for children, adolescents, and adults.

Another potential limitation of the present study is the specificity with which we can identify age-related effects, which examined emotional interference across three age-defined groups. As such, a longitudinal design is needed to assess for dimensional changes in interference to socially- vs. socially- and emotionally-salient distractor stimuli. Our cross-sectional sample was also not designed to capture the extent to which age-related differences in the child and adolescent groups could be attributed to puberty. Given findings that puberty may contribute to increased reactivity to emotionally-salient information stimuli in adolescents, examination of the specific effects of puberty on performance in anxious youth may further our understanding of how developmental processes influence the subcomponents of cognitive control that have been implicated in anxiety disorders. Likewise, an examination of interference *specific* to task-irrelevant emotional information on the EIDF task in other clinical populations would be important to identify the aspects of socially- and emotionally-relevant information that are most distracting—and therefore indicative—of the pathophysiology for other affective disorders. For example, as suggested by Manelis et al. (2015), individuals who have increased familial risk for bipolar disorder show greater interference to happy vs. negative faces as well as altered functional connectivity between the amygdala and frontal cortex regions. Furthermore, the role that flexibility in cognitive control might play in the etiology of affective disorders should be further explored by comparing the degree to which individuals at low vs. high risk for affective disorders show improvements in task performance when distractor stimuli are at spatially-relevant vs. irrelevant locations. Finally, results from the present study should be replicated with a larger sample size but nevertheless imply that the inclusion of highly-salient *target* stimuli, like dynamically-changing facial expressions, could be beneficial for understanding the conditions whereby adolescents show interference from task-irrelevant information.

In summary, findings from the current study suggest that whereas children and adults showed interference (slowed correct responses) to socially-salient distractor stimuli, adolescents did not. Although research generally suggests that adolescents' heightened reactivity to emotional information contributes to the curvilinear development of emotional interference, these findings suggest that there are certain contexts in which adolescents might exhibit better regulation of interference to socially-salient distractor stimuli, than adults or children. It is therefore important to develop experimental cognitive control paradigms that vary in affective demands in order to advance knowledge about cognitive-affective vulnerability markers. That is, this type of approach can elucidate the complex ways in which increased sensitivity to socio-emotional information could serve as both a risk and protective factor at different developmental periods. In addition, consistent with models highlighting the role of cognitive biases in the development of anxiety symptoms and disorder, we found that dynamically-emerging angry faces produce greater interference for high trait-anxious individuals who also have low levels effortful control. These findings provide further evidence for etiological pathways by which the combination of high trait anxiety and low effortful control increases vulnerability for anxiety disorders across development.

## Author contributions

JS, RD, and CL developed the initial study concept and design. DK developed the EDIF experimental task. PT and CL developed study hypotheses. PT had primary responsibility for drafting the paper. All authors revised the manuscript and approved the final version for submission.

### Conflict of interest statement

The authors declare that the research was conducted in the absence of any commercial or financial relationships that could be construed as a potential conflict of interest.

## Footnotes

^1^Results from repeated measures ANOVA analyses of accuracy and correct RTs remain unchanged when excluding the adult participant who was 52-years-old (see Supplementary Information). Thus, we report results from the full sample.
