# Supplementary material for: Age-Related Developmental and Individual Differences in the Influence of Social and Non-social Distractors on Cognitive Performance
Source: Front Psychol. 2018 Jun 8;9:863. doi: 10.3389/fpsyg.2018.00863 (PMC6002742; doi:10.3389/fpsyg.2018.00863)
Supplement: Supplementary file 1 [file Table_1.PDF]

Supplementary Information

To ensure that findings were not due to an outlier in our adult age group, we re-ran repeated measures ANOVAs examining the effects of distractor type and age group on (1) accuracy, (2) correct reaction time, and (3) difference scores, i.e., EmoShapeDiff and EmoIdentDiff scores without the participant who as 52 year olds. The models were constructed in a manner that was identical to the data analytic plan described in the main analyses.

Developmental Differences in Accuracy and Reaction Time: Repeated Measures ANOVA. Results from a 6 (Distractor Type) × 3 (Age Group) mixed ANOVA with repeated measures revealed a main within-subjects effect of Distractor Type on accuracy, $F(10,38)=4.37$, $p=.000$, $\eta^2=.10$. As seen in Figure 2, Tukey HSD-adjusted post-hoc tests indicated that participants were more accurate on trials that included socially-salient distractor stimuli (both emotional-social and neutral-social distractor types) than on trials that included distractors with low social and emotional saliency (non-social/emotional distractor), all *p's*<.020. There were no significant age-related between-subjects effects on accuracy, $F(2,42)=2.35$, $p=.11$, $\eta^2=.10$ nor was there a significant Distractor Type × Age Group interaction effect, $F(10,78)=.58$ $p=.83$, $\eta^2=.03$.

Results from a second 6 (Distractor Type) × 3 (Age Group) repeated measures ANOVA modeling correct reaction times (correct RTs) revealed no main within-subjects effect of Distractor Type, $F(5,38)=1.27$, $p=.28$, $\eta^2=.03$. There was, however, a significant main between-subjects effect of age, $F(2,42)=8.60$, $p=.003$, $\eta^2=.28$. Tukey HSD-adjusted post-hoc tests indicated that children were significantly slower on correct trials than both adults and adolescents, all *p's*<.02. Adolescents and adults did not show a significant difference on correct $p=.222$. Finally, there was no significant Distractor Type × Age Group interaction effect, $F(10,78)=1.41$, $p=.18$, $\eta^2=.06$.

Developmental Differences in Interference from Emotional-social vs. Non-social/emotional Distractors: Repeated measures ANOVA modeling EmoShapeDiff scores. Results from a 4 (Difference Score Type) x 3 (Age Group) ANOVA with repeated measures indicated that interference to social-emotional (emotional faces) vs. non-social/emotional (shape) distractors significantly differed by age. Specifically, there was a significant main between-subjects effect of age, $F(2,42)=6.39$, $p=.004$, $\eta^2=.23$. Tukey HSD-adjusted post-hoc tests indicated that children and adults showed a similar degree of interference, $p=1.00$. However, adults showed more interference from social-emotional distractors than adolescents, $p=.017$. Likewise, children also showed more interference from social-emotional distractors than adolescents, $p=.016$. There was no significant main within-subjects effect of Difference Score Type, $F(3,40)=1.79$, $p=.152$, $\eta^2=.04$, suggesting that 'interference' due to the presence emotional-social distractors, that is, changes in reaction time associated with the presence of emotional-social distractors, did *not* differ across specific (angry, fearful, sad, happy) emotions. There was also no significant Difference Score Type × Age Group interaction effect, $F(6,82)=.07$, $p=.999$, $\eta^2=.004$.

Developmental Differences in Interference from Emotional- vs. Neutral-Social Distractors: Repeated measures ANOVA modeling EmoIdentDiff scores. Results from a 4 (Difference Score Type) × 3 (Age Group) repeated measures ANOVA modeling EmoIdentDiff

scores were conducted to test whether emotional salience of faces is associated with cognitive interference. Results revealed no significant main within-subjects effects of Difference Score Type, $F(3,40)=1.79$, $p=.152$, $\eta^2=.04$, or between-subjects effects of Age Group, $F(2,42)=.04$, $p=.95$, $\eta^2=.002$. In addition, there was no significant Difference Score Type × Age Group interaction effect, $F(6,82)=.07$, $p=.999$, $\eta^2=.03$.

Supplementary Table 1. Summary of Regression Models for Emotional Interference Difference Scores.

	β	*SE* (B)	*t*	R^2	*F*	*df*	*p*
Anger (Anger RT – Shape RT)				.27	3.64	(4,39)	.013
Age	.04	1.36	.31				
Trait Anxiety	.22	7.55	1.50				
Effortful Control	$-.29^{\dagger}$	7.47	-1.96				
Trait Anxiety X Attentional Control	$-.31^{*}$	7.75	-2.19				
Fear (Fearful RT – Shape RT)				.11	.1.23	(4,39)	.31
Age	-.12	1.68	-.72				
Trait Anxiety	$.35^{*}$	9.27	2.11				
Effortful Control	.11	9.18	.69				
Trait Anxiety X Attentional Control	-.03	9.52	.19				
Happy (Happy RT – Shape RT)				.01	.13	(4,39)	.97
Age	-.88	1.77	-.54				
Trait Anxiety	.04	9.90	.21				
Effortful Control	-.03	9.80	-.15				
Trait Anxiety X Attentional Control	.02	10.16	.44				
Sad (Sad RT – Shape RT)				.05	.52	(4,39)	.72
Age	.07	1.52	.45				
Trait Anxiety	.10	8.35	.56				
Effortful Control	-.03	8.30	-.15				
Trait Anxiety X Attentional Control	-.19	8.62	1.18				

Supplementary Table 1. Summary of Regression Models for Emotional Interference Difference Scores. Linear regression models were used to test for associations between emotional interference and participant age (in years), trait anxiety (z-scored), temperamental effortful control (z-scored), and interaction between trait anxiety and effortful control. Separate models were conducted to investigate individual differences in interference to anger, fearful, happy, and sad distractors (i.e., dynamically-emerging facial expressions). All predictor variables were centered.

Notes. *$p < .05$. ⱡ$p < .10$.
